# Outcome and the effect of age and socioeconomic status in 1318 patients with synovial sarcoma in the English National Cancer Registry: 1985–2009

**DOI:** 10.1186/s13569-016-0058-y

**Published:** 2016-10-14

**Authors:** Bernadette Brennan, Charles Stiller, Robert Grimer, Nicola Dennis, John Broggio, Matthew Francis

**Affiliations:** 1 Department of Paediatric Oncology, Royal Manchester Children’s Hospital, Oxford Road, Manchester, M13 9WL UK; 2 Knowledge and Intelligence Service West Midlands, Public Health England, Birmingham, UK; 3 National Cancer Registration and Analysis Service, Public Health England, Birmingham, UK; 4 Department of Orthopaedic Oncology, Royal Orthopaedic Hospital, Birmingham, UK

**Keywords:** Synovial sarcoma, Age, Social deprivation

## Abstract

**Background:**

The role of age as a prognostic factor has been examined in single institutional studies and in larger data sets from the SEER database, showing a survival advantage for younger versus adult patients with synovial sarcoma (SS). To further assess the role of age, socioeconomic status and other prognostic factors on outcome for SS, we analysed a contemporary all-age population-based cohort of patients with SS registered in England.

**Methods:**

The data on 1318 synovial sarcomas diagnosed in England between 1985 and 2009 were retrospectively analysed for incidence, and the effect of age, patient characteristics and deprivation on outcome using both univariate and multivariate analysis.

**Results:**

The incidence of SS increased to 1.4 per million over the time period, the numbers diagnosed in patients under 10 years of age were small. The site or incidence of metastases did not vary between age groups. There were, however, significant differences (p < 0.05) in the 5-year relative survival rates between patients aged 0–19 years and those ≥20 years of age, 76 % and 53 % respectively. Survival was better in localised tumours at an extremity site. In multivariate analysis higher mortality occurred in older patients, non-extremity site, presence of metastases, female adults and a higher deprivation score.

**Conclusions:**

Synovial sarcoma in children/teenagers compared with adults, have a similar clinical presentation in this population-based series, but a superior outcome. The finding of socioeconomic deprivation affecting outcome in SS needs further exploration in a complete and contemporary dataset, where all prognostic variables are present.

## Background

In childhood and adolescence synovial sarcoma (SS) is rare with an annual incidence of 0.5 per million and only six new cases per year diagnosed in those less than 15 years of age in the UK [1]. The incidence, however, of SS differs across age groups. In the North American population-based surveillance epidemiology, and end results (SEER) cancer registry, the age-standardised incidence of SS was higher in adults at 1.42 per million [2] versus 0.81 per million in patients under 19 years of age [2]. Recorded incidence may have changed over time due to more accurate diagnosis, with the recognition of a characteristic translocation involving chromosome 18 and X, resulting in the detection of one of several types of fusion genes (SYT-SSX1, 2 and 4) in 90 % of cases [3, 4].

Surgery remains an important aspect of treatment for localised SS with radiation, which may facilitate surgical resection [5]. In paediatric practice, less radiotherapy is used in view of late effects [21]. The role of chemotherapy in SS is not completely defined, but is used more in paediatric patients [6, 7]. In adults, chemotherapy is not standard of care, particularly in localised disease regardless of tumour size, but is used in high-risk patients and those with metastases [8] or as part of investigational trials [9].

The role of age as a prognostic factor has been examined in single-institutional studies and the SEER database; Ferrari et al. demonstrated that children had a better outcome than adults, asserting but not proving that this was due to the use of more chemotherapy [10]. Socioeconomic status has been shown to be a significant predictor of outcome in lung, colon and breast cancer, with those patients with lower socioeconomic status having a worse outcome [11–13]. The effect of socioeconomic status on the outcome of SS has not been studied. To further assess the role of age, socioeconomic status and other prognostic factors on outcome for SS, we analysed a contemporary all-age population-based cohort of patients with SS registered in England.

## Methods

This study carried out a retrospective population-based analysis of the incidence, clinical characteristics, and survival of SS.

### Source of data

The anonymised data were extracted from the National Cancer Data Repository (NCDR), a compilation of data collected by all regional cancer registry offices in England. The information includes age, sex, ethnicity, post code of residence, anatomical site of tumour, and some treatment data. Two different versions of the NCDR were utilised for the analyses. Incidence and survival data was utilised from the 1985–2009 version of the NCDR—containing 1318 SS. The 1990–2010 NCDR was utilised so that treatment data could be assessed as recently as possible, with 70 SS diagnosed in 2010.

All SS diagnosed in England between 1985 and 2009 classified by the 10th revision of the International Classification of Diseases (ICD-10 site code) and International Classification of Disease for Oncology, version 3 (ICD-0-3) morphology codes M9040-9043 were analysed [14, 15]. All incidence cases identified were included in the analysis except for four death certificate only (DCO) cases which were excluded for the purpose of survival analyses. This is in line with the recommended practice of data quality for conducting survival analyses [28] and none of the other quality controls were relevant to this cohort. Death certificates are provided by the Office of National Statistics (ONS).

Morphology subtype was not analysed since 1107 (84 %) of the 1318 patients were recorded as having SS not otherwise specified (NOS) [M9040].

Hospital Episode Statistics (HES) data is a record of all hospital admissions. HES data captures information on admission date, operations and procedures, and other co-morbidities. HES data was linked to the NCDR on the patient demographics—NHS number, date of birth and postcode.

Patient ethnicity is described in the analyses although not included in any statistics due to the incompleteness of this data item.

Metastatic status was derived from the HES data If a patient had a diagnosis of ICD-10 codes C77 (Secondary and unspecified malignant neoplasm of lymph nodes), C78 (Secondary malignant neoplasm of respiratory and digestive organs) or C79 (Secondary malignant neoplasm of other sites) recorded during an admission within 4 months following sarcoma diagnosis. OPCS Classification of Interventions and Procedures, version 4 (OPCS4) codes were extracted from the HES data for SS patients. Surgery was classified as the OPCS4 codes which relate to radical curative surgery, and occurred within 6 months of initial diagnosis. Patients for which no HES record could be identified were removed for this set of analyses. This resulted in 776 cases out of 819 diagnosed between 2000 and 2010 available for analysis.

Due to the low incidence of SS occurring outside of the extremities, cancer sites were classified as extremity or other for the purpose of statistical modelling.

### Socioeconomic status

Socioeconomic status was assessed using the income domain (ID) of the index of multiple deprivation 2010 (IMD) [16]. The IMD uses information from the National Census to form a score from 7 measures of deprivation for a given area: income, employment, health, education, crime, access to services, and living environment. The scores are assigned to geographic areas within England, each with a local population of approximately 1500 so that each postal code can be allocated a score. The scores range from 1 (least deprived) to 5 (most deprived). For reasons of comparability with other parts of the United Kingdom, cancer data is analysed with respect to the income domain only.

### Statistical methods and data analysis

Confidence intervals around incidence rates were calculated using the gamma method [17]. Significant differences for the incidence rates were inferred by comparing the confidence intervals (CI) for different rates. If the confidence intervals overlapped then the rates are not significantly different and if the confidence intervals did not overlap then the rates are significantly different. A p value of <0.05 was considered significant. All statistical tests were two-sided.

Relative survival was defined as the observed survival in the patient group divided by the expected survival of the general population, matched by age, sex and calendar year. Relative survival was estimated by the Ederer II method [18] using the STRS programme in STATA (version 13). National life tables were obtained from the Cancer Research UK Cancer Survival Group at the London School of Hygiene and Tropical Medicine. All patients were last followed up on 31 December 2013 and analysed in 2014. Therefore, patients diagnosed in 2009 were excluded from survival analyses because of lack of 5-year follow-up. Five-year relative survival was calculated using 5-year rolling averages. Cox proportional-hazard models were used to identify the prognostic factors most likely to result in death for people with SS and were calculated for patients diagnosed between 2000 and 2010. To assess the role of age as a prognostic factor a cut off of 19 years was chosen in view of the data from the SEER analysis (2).

Multivariable logistic regression models were used to assess the risk of metastases dependent on age at diagnosis, sex, site and ID score.

## Results

### Patient characteristics

**Fig. 1:**
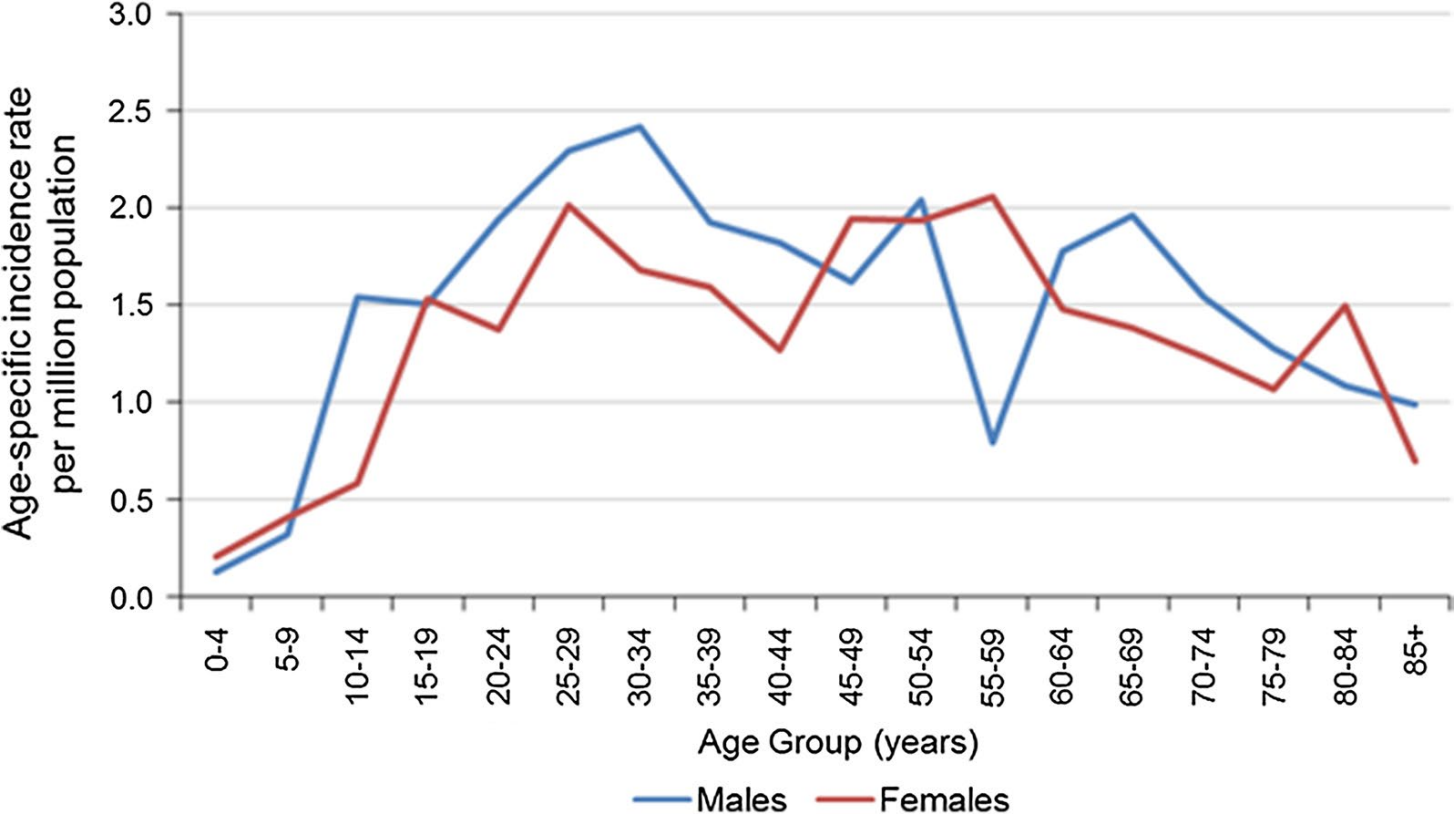
Age specific incidence rates males and females, for the years 1985–2009 in England

Between 1985 and 2009, 1318 SS were registered. Age at diagnosis ranged from 8 months to 93 years, with only 25 cases reported under the age of 10 years. The largest numbers and highest age-specific rates occurred in males aged 30–34 years. (Fig. 1).

The age-standardised incidence rates (3-year rolling average) for all ages combined increased from 0.7 per million to 1.4 per million in males and from 0.5 per million to 1.4 per million in females between 1985 and 2009, with the steepest increase from 1997 onwards.

Among the population between 0 and 19 years the age-standardised incidence rates fluctuated between 0.08 per million and 1.1 per million. However, in the 20-years and over age group there was a statistically significant increase from 0.7 per million in 1985 to 1.4 per million in 2009 (p < 0.05).

Table 1 describes demographic and clinical characteristics for all ages combined and for the two age groups of 0–19 years and ≥20 years. Univariate analysis of the clinical characteristics in Table 1 showed there were no differences between children/adolescents and adults (all p values >0.07). The commonest site was extremity (65 %) followed by soft tissues of the trunk.

**Table 1:** Patient characteristics in 1318 patients with synovial sarcoma

	All patients		Children and teenagers		Adults	
	No.	%	No	%	No.	%
Total	1318	–	182	–	1136	−
Age (years)						
0–9	25	2	25	14	0	0
10–19	157	12	157	86	0	0
20–29	237	18	0	0	237	21
30–39	239	18	0	0	239	21
40–49	207	16	0	0	207	18
50–59	189	14	0	0	189	17
60–69	130	10	0	0	130	11
≥ 70	134	10	0	0	134	12
Sex						
Female	628	48	79	43	549	48
Male	690	52	103	57	587	52
Location						
Head and neck	66	5	14	8	52	5
Extremity	861	65	114	63	747	66
Lungs and pleura	60	5	6	3	54	5
Trunk	190	14	27	15	163	14
Others	141	11	21	12	120	11
Quintile of the income domain of the index of multiple deprivation 2010						
Least deprived	257	19	36	20	221	19
2	256	19	30	16	226	20
3	261	20	29	16	232	20
4	278	21	41	23	237	21
Most deprived	266	20	46	25	220	19
Stage at diagnosis^a^						
Total known	776		109		667	
Localised	656	85	97	89	559	84
Metastatic	120	15	12	11	108	16
Did patients have surgery?						
Surgically treated	550	71	84	77	466	70
Not surgically treated	226	29	25	23	201	30

There were no significant differences in the proportions of children or adults having any of the tabulated characteristics

^a^Based on 2010 data to allow further inclusion of metastases

Among 776 of 819 patients diagnosed between 2000 and 2010 for which HES records were identified, whether distant metastases were found at diagnosis and whether the patient had any surgical intervention in the month preceding diagnosis or the 6 months following diagnosis were analysed, but there were no significant differences between the two age groups for either distant metastases or surgical intervention (p > 0.1) (Table 1).

The income domain of the IMD scores were equally distributed in the 5 groups, with a non-significant higher percentage in the most deprived group (score 5) for the 0–19 years cohort (all p values >0.06).

### Survival and prognostic factors

**Fig. 2:**
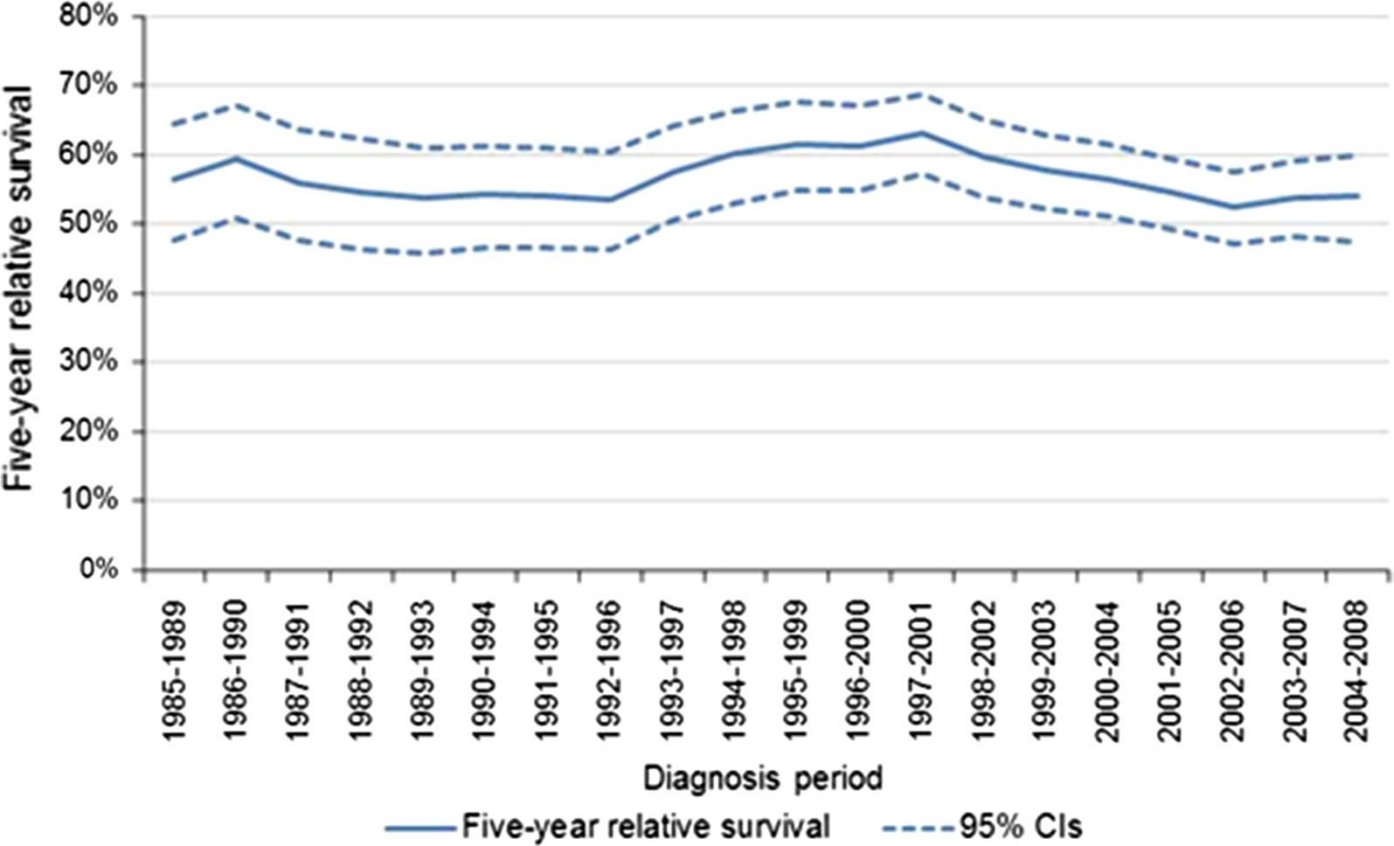
Synovial sarcoma 5-year relative survival rates (5-year rolling average) (England: 1985–2008). *LCI* lower confidence interval, *UCI* upper confidence interval

**Fig. 3:**
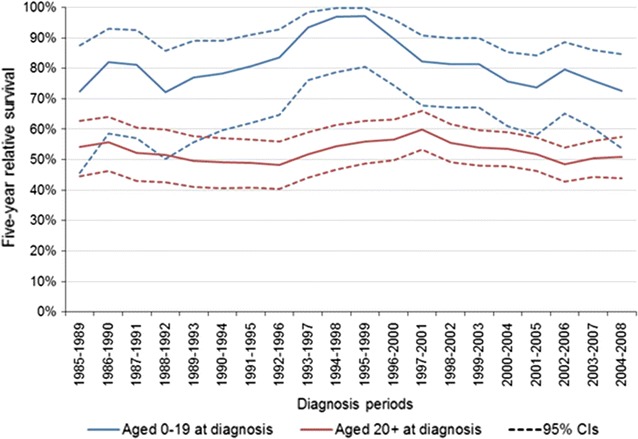
5-year relative survival rates for synovial sarcoma (5-year rolling average) for the years 1985–2009 in England 0–19 years and 20+ years

There was no significant variation in the 5-year relative survival rates for SS over the period 1985–2008, regardless of age groups. (Figs. 2, 3). The 5-year relative survival rate was 56 % [CI: (47–64 %)] for patients diagnosed between 1985 and 1989, and 56 % [CI: (51–61 %)] in the most recent diagnosis years (2004–2008). There were, however, significant differences (p < 0.05) in the 5-year relative survival rates between patients aged 0–19 years and those ≥20 years of age, both in years 1985–89 [72 %, 95 % CI: (46–88 %)] vs. [53 %, 95 % CI: (44–62 %)] and in years 2004–08 [76 %, 95 % CI: (63–85 %)] vs. [53 %, 95 % CI: (47–58 %)]. (Fig. 3).

There was no difference in outcome among sex and ID of IMD. There was, however, a worse outcome for tumours at a non-extremity site compared to tumours of the extremity, 5-year relative survival 34 % [95 % CI: (27–42 %)] and 71 % [95 % CI: (64–76 %)] respectively, p < 0.001. Metastatic spread was also associated with a poorer outcome, 5-year relative survival was 7 % [95 % CI: (2, 15 %)] for those with distant metastases and 65 % [95 % CI: (59, 70 %)] for those without, p < 0.001.

For all patients in the multivariate analysis, age remained significant; the highest mortality was in those ≥70 years of age with a hazard ratio (HR) of 8.48 relative to those aged between 0–19 years (Table 2). For the whole population other factors associated with a higher mortality included non-extremity tumour site, female sex, presence of distant metastases and ID score-5 (Table 2). For those <20 years of age non-extremity site lost significance but the presence of distant metastases remained significant. For adults (≥20 years), male sex, presence of distant metastases and non-extremity site remained significant, with ID of IMD score only just significant. (Table 2) It is noteworthy that the number of children/teenagers <20 years of age was small in this model with only 182 in total. We explored possible associations with the age at diagnosis and the ID score but did not find any significant results in several different groupings of patient age at diagnosis.

**Table 2:** Cox proportional hazards regression for mortality in 819 patients with synovial sarcoma diagnosed between 2000 and 2010

Variable	All patients	Children and adolescents	Adults
	Hazard ratio (95 % CI)	Hazard ratio (95 % CI)	Hazard ratio (95 % CI)
Age category, years			
0–19	1.00	–	–
20–29	1.39 (0.84–2.31)	–	–
30–39	1.92 (1.20–3.07)**	–	–
40–49	2.12 (1.32–3.43)**	–	–
50–59	2.73 (1.68–4.45)***	–	–
60–69	4.98 (3.08–8.05)***	–	–
≥ 70	8.48 (5.27–13.64)***	–	–
Sex			
Female	1.00	1.00	1.00
Male	0.67 (0.54–0.84)***	0.95 (0.40–2.23)	0.70 (0.56–0.88)**
Primary site			
Extremity	1.00	1.00	1.00
Other	2.25 (1.79–2.82)***	1.73 (0.68–4.36)	2.23 (1.76–2.82)***
Stage at diagnosis^a^			
Localised	1.00	1.00	1.00
Metastatic	6.15 (4.75–7.95)***	42.4 (12.53–143.70)***	4.86 (3.74–6.33)***
Quintile of the income domain of the index of multiple deprivation 2010			
Least deprived	1.11 (0.78–1.59)	2.39 (0.47–12.19)	1.17 (0.81–1.68)
2	1.34 (0.95–1.91)	1.68 (0.31–9.16)	1.33 (0.93–1.90)
3	1.00	1.00	1.00
4	1.18 (0.83–1.68)	0.61 (0.13–2.83)	1.22 (0.84–1.76)
Most deprived	1.48 (1.03–2.12)*	2.35 (0.54–10.16)	1.27 (0.88–1.84)
Time varying covariate			
Primary site	0.76 (0.64–0.91)**	–	0.73 (0.61–0.88)**

* Statistically significant at p < 0.05

** Statistically significant at p < 0.01

*** Statistically significant at p < 0.001

^a^Based on 2010 data to allow further inclusion of metastases

For patients with extremity SS, they were less likely to have distant metastases at diagnosis than those with SS at other sites with an odds ratio of 0.67 (p < 0.05). This was the only characteristic associated with a decreased risk of metastases (Table 3).

**Table 3:** Multivariable logistic regression to assess the relative risk of being diagnosed with distant metastases dependent on age at diagnosis, sex, site and ID score in 819 patients with synovial sarcoma diagnosed between 2000 and 2010

Variable	Odds ratio (95 % CI)
Age 20–29	1.31 (0.60–2.83)
Age 30–39	2.14 (1.03–4.43)*
Age 40–49	1.20 (0.55–2.65)
Age 50–59	1.68 (0.76–3.71)
Age 60–69	1.57 (0.67–3.67)
Age 70+	1.52 (0.64–3.62)
Sex (male)	0.72 (0.49–1.08)
Primary site (extremity)	0.66 (0.49–0.98)*
Quintile of the income domain of the index of multiple deprivation 2010 (1)	0.76 (0.41–1.41)
(2)	0.71 (0.38–1.32)
(4)	0.93 (0.51–1.72)
(5)	0.87 (0.47–1.61)
Constant	0.20 (0.09–0.43)***

* Statistically significant at p < 0.05

*** Statistically significant at p < 0.001

## Discussion

This analysis of 1318 patients with SS diagnosed from 1985 to 2009 is the largest population-based study to date, including all stages of disease. The unusually large numbers for such a rare tumour allowed us to study changes in incidence and outcome, the effect of prognostic factors, and uniquely the effect of severe deprivation as represented by the ID of the IMD score on outcome. We confirmed that age is an important prognostic factor, with patients aged 0–19 years having a significantly better outcome but severe deprivation does affect outcome. As significant is the outcome for elderly patients the highest mortality was in those ≥70 years of age. Furthermore, the value of these findings is enhanced due to the quality of the national registrations and the high level of case ascertainment with minimal serious errors detected on regular completeness and validity checks [19].

There are, however, limitations to this study. Although data are available on the presence of metastases or not at diagnosis, which was similar in the two age groups, 0–19 years and ≥20 years, other factors explaining the better outcome in the younger age group, are missing. Data on tumour size, biology, chemotherapy and radiotherapy, were not routinely collected by the cancer registries in England until 2013. Therefore, the better outcome in children and adolescents maybe due to other factors unavailable in this series. Lastly, 77 % of children/adolescents and 70 % of adults were recorded as having surgery, compared with 98 and 88 % respectively in the SEER study [2], suggesting that surgery is incompletely recorded.

Two roughly contemporary Italian hospital-based series have contrasting results. At the Instituto Tumori, Milan, among the 255 patients with localised disease, more chemotherapy was given to younger patients suggesting that this accounted for their better outcome [10]. At Rizzoli, however, among patients with localised disease there was no difference in chemotherapy usage between age groups, but children had better survival than adults [22]. The lack of effect of treatment in particular chemotherapy has also been confirmed in a recent Netherlands cancer registry study in localised SS, furthermore they demonstrate that younger age is again important for outcome regardless of site or tumour size. [25].

The better outcome in children/adolescents compared to adults with SS was also found in the analysis of the SEER data [2]. Other similarities in our study included similar clinical presentation between adults and children/adolescents in terms of metastases and the site of the tumour. Our study also confirmed that those with limb primaries and no metastases had a superior outcome [2]. Poor outcome in SS arising in non-limb sites has also been demonstrated in a smaller Italian series and the SIOP MMT analysis [20, 21]. A possible explanation for the better outcome in younger patients with SS is biology, in particular the role of genomic index (GI) [26]. Increasing genomic instability as scored by GI is more frequent in adults compared to children with SS, and predicts for metastatic relapse and survival [26]. Furthermore GI is independent of response to neoadjuvant chemotherapy and remains an independent prognostic factor [27]. This factor could not be analysed in our series, and needs confirming in a prospective trial.

The overall 5-year relative survival at 57 % compared favourably with similar population-based series, in particular the SEER database [2]. Compared with series, which are institutionally based, our outcome seems superior, but it is likely that institutional series reflect a higher risk group, and contains patients from an earlier time period [10, 22]. It is disappointing that over time the survival in England has not improved. Recent paediatric series from the European Paediatric soft tissue group have a much better outcome, but they only included young patients with localised disease [24].

A novel feature of this study was the ability to assess the role of deprivation on outcome using the income domain of the Indices of Multiple Deprivation 2010. ID scores have been widely used to study the relationship between socio-economic factors and outcome in cancer [11–13]. These studies demonstrated a worse outcome among patients with more deprivation and higher deprivation scores. For the whole cohort in our study, patients with the highest ID of IMD score 5 only, and hence the most deprived, had a worse outcome, this held in the adult cohort but was not significant in the younger age group, possible due the small numbers <19 years of age. This may be plausibly due to diagnostic delays (professional or patient), or to poor awareness of this tumour, but the lack of data on tumour size, treatment received and length of clinical pathway hampers any further interpretation. However, the ID score did not predict for increased metastases at diagnosis. The role of deprivation on survival from SS has not been studied previously, but in Ewing sarcoma survival was significantly lower in deprived groups [23].

## Conclusion

Children/teenagers and adults with SS have a similar clinical presentation but children/teenagers have a superior outcome. The lack of complete data on treatment received makes it impossible to tell whether this or biological variables explain this difference, though the latter is more likely. The finding of severe socioeconomic deprivation as presented as the ID of IMD score affecting outcome in SS needs further exploration in a complete and contemporary dataset, where all prognostic variables are present. This may be possible in the current national cancer registry. The paucity of all-ages, large and international randomised studies in SS, with the testing of new targeted agents, may have contributed to the static outcome in this cohort over this time period.

## Abbreviations

CI: confidence intervals
DCO: death certificate only
GI: genomic index
HES: hospital episode statistics
ID: income domain
IMD: index of multiple deprivation
NCDR: national cancer data repository
NOS: not otherwise specified
ONS: office of national statistics
SEER: surveillance epidemiology and end results
SS: synovial sarcoma
STS: soft tissue sarcoma
